# Metabolomics Characterization of Phenolic Compounds in Colored Quinoa and Their Relationship with In Vitro Antioxidant and Hypoglycemic Activities

**DOI:** 10.3390/molecules29071509

**Published:** 2024-03-28

**Authors:** Ling Zhang, Bin Dang, Yongli Lan, Wancai Zheng, Jiwei Kuang, Jie Zhang, Wengang Zhang

**Affiliations:** 1 Laboratory for Research and Utilization of Qinghai Tibet Plateau Germplasm Resources, Qinghai University, Xining 810016, China; 13897604262@163.com (L.Z.); 2008990019@qhu.edu.cn (B.D.); 13565849218@163.com (W.Z.); 2023990011@qhu.edu.cn (J.K.); 2015990070@qhu.edu.cn (J.Z.); 2 Key Laboratory of Qinghai Province Tibetan Plateau Agric-Product Processing, Qinghai University, Xining 810016, China; 3 College of Food Science and Engineering, Northwest A & F University, Yangling 712100, China; yonglilan@nwsuaf.edu.cn

**Keywords:** *Chenopodium quinoa* Willd., phenolic compounds, metabolomics, antioxidant activity, α-amylase, α-glucosidase

## Abstract

*Chenopodium quinoa* Willd. is rich in phenolic compounds and exhibits diverse biological activities. Few studies have focused on the effect of colored quinoa’s phenolic profile on potential biological activity. This study used a UPLC–MS/MS-based metabolomic approach to examine the quinoa phenolics and their association with in vitro antioxidant and hypoglycemic properties. In total, 430 polyphenols, mainly phenolic acids, flavonoids, and flavonols, were identified. Additionally, 121, 116, and 148 differential polyphenols were found between the white and black, white and red, and black and red comparison groups, respectively; 67 polyphenols were screened as shared key differential metabolites. Phenylalanine, tyrosine, and the biosynthesis of plant secondary metabolites were the main differently regulated pathways. Black quinoa had better total phenolic contents (643.68 mg/100 g DW) and antioxidant capacity, while white quinoa had better total flavonoid contents (90.95 mg/100 g DW) and in vitro α-amylase (IC50 value of 3.97 mg/mL) and α-glucosidase (IC50 value of 1.08 mg/mL) inhibition activities. Thirty-six polyphenols, including epicatechin and linarin, etc., were highly correlated with in vitro antioxidant activity, while six polyphenols, including tiliroside and chrysoeriol, etc., were highly correlated with in vitro hypoglycemic activity. This study may provide important information for colored quinoa resources to develop their healthy food applications.

## 1. Introduction

*Chenopodium quinoa* Willd. is a dicotyledonous pseudocereal of the genus Quinoa in the family Amaranthaceae. It contains rich unsaturated fatty acids, amino acids, dietary fiber, protein, betaine, minerals, polyphenols, and other active ingredients [1], which can be used as an ideal dietary supplement to improve human health. Quinoa seeds possess a variety of grain colors, with red, black, and white genotypes being the most common. It has been shown that quinoa’s seed color is intricately linked to its chemical content. Generally, the darker the seed color, the higher its nutritional value [2]. Therefore, colored quinoa is considered a valuable new class of cereal resource that has received increasing attention in the food industry in recent years.

Polyphenols are a group of secondary metabolites that have several physiological properties, including antibacterial, antitumor, blood glucose regulation, anti-inflammatory, and antioxidant properties, and are essential for the health benefits and grain color variation of quinoa [3,4,5]. Currently, quinoa has been shown to contain around 20 distinct phenolic compounds, the bulk of which are phenolic acids, including ferulic acid, vanillic acid, caffeic acid, and their derivatives [6], whereas flavonoids are primarily present in the glycoside form of quercetin and kaempferol [7]. In addition, black quinoa is generally considered to have a richer phenolic substance and better biological activity [8,9]. With the development of modern analytical techniques, more investment has been made in the identification of the common polyphenols in quinoa, but the comprehensive exploration of the quinoa polyphenol profile is limited. This might not be conducive to understanding the material basis of various physiological functions of quinoa polyphenols.

Widely targeted metabolomics based on LC–MS combines the best features of both untargeted and targeted metabolomics, which is ascendant for the analysis of secondary metabolites and their metabolic pathways in crops [10,11]. At present, reports have characterized the chemical components of quinoa using widely targeted metabolomics techniques [12,13]. However, the shortcoming is that the comprehensive identification and comparative analysis of phenolic compounds in colored quinoa, as well as the relationship between phenolic compounds and in vitro antioxidant and hypoglycemic activities, is still insufficient.

With that in mind, this study was conducted to measure the phenolic compounds of quinoa with different grain colors based on an ultra-performance liquid chromatography–tandem mass spectrometry (UPLC–MS/MS), employing a widely targeted metabolomics approach. The differential metabolism of polyphenols and their metabolic pathways were determined using multivariate statistical methods. Subsequently, the polyphenol contents of three colored quinoa extracts and their in vitro antioxidant capacity and α-amylase and α-glucosidase inhibitory activities were comparatively analyzed. Our objective was to define the effect of grain color on the quinoa polyphenol profile and to preliminarily disclose the possible antioxidant and glucose-regulating polyphenol. This study may offer a theoretical foundation for the utilization of quinoa polyphenol resources in health foods.

## 2. Results and Discussion

### 2.1. Metabolomics Profiling of Three Different Colored Quinoa

On a UPLC–MS/MS system, samples were analyzed in both negative and positive ESI modes. Total ion current (TIC) analysis of QC samples was used to check the consistency of metabolite extraction and detection. The TIC curves were overlaid with the metabolite detection results (Figure S1A,B). In addition, Pearson correlation coefficients were determined in duplicate intra-group samples, ensuring good homogeneity (R^2^ close to 1).

### 2.2. Polyphenol Identification of Three Different Colored Quinoa

**Figure 1:**
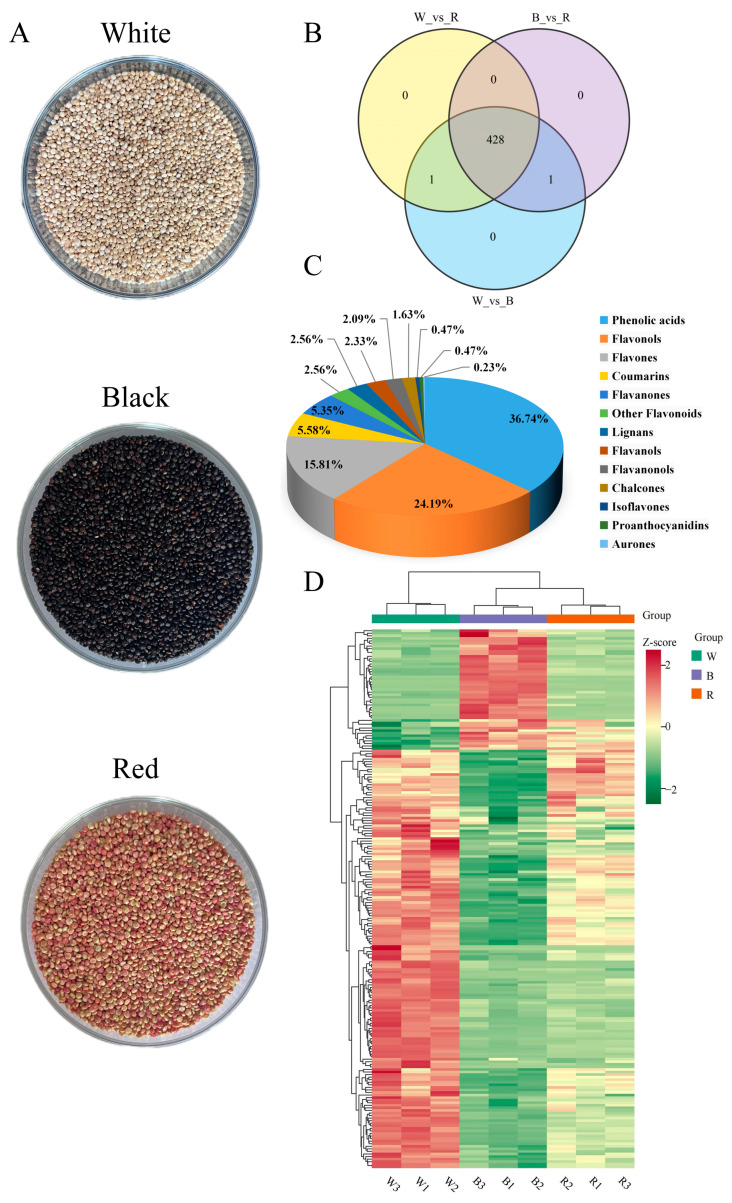
Overview of the detected polyphenol metabolites in white, black, and red quinoa seeds. (**A**) Appearance of the three quinoa seeds. (**B**) The overlapping and unique metabolites among the three quinoa seeds. (**C**) Classification of the polyphenol metabolites in the three quinoa seeds. (**D**) Hierarchical cluster analysis (HCA) results of metabolite variation between and within groups.

The multipeak detection plots of the metabolites in MRM mode and the basic information of the identified chemicals are shown in Figure S1C,D and Table S1, respectively. Three different colored quinoa seeds are exhibited in Figure 1A. A total of 430 polyphenols were identified in three different colored quinoa samples (Figure 1B), including 158 phenolic acids (36.74%), 235 flavonoids (54.65%), 24 coumarins (5.58%), 11 lignans (2.56%), and 2 proanthocyanidins (0.47%) (Figure 1C). Apigenin-6-C-(2″-glucuronide)xyloside is a monomeric phenol unique to B. Lignan-7-*O*-glucoside-5-*O*-arabinoside is the monomeric phenol unique to W. The main polyphenol metabolites are flavonols, flavonoids, and phenolic acids. Based on the relative contents of phenolics, 30 phenolic compounds with high contents were screened (Figure S2), with significant differences found in the distribution of these compounds between groups. As the main phenolic acids and flavonoids in quinoa, vanillic acid, protocatechuic acid, ferulic acid, isoquercitrin, kaempferol, quercetin, neohesperidin, hesperidin, and hydroxybenzoic acid have been reported in the literature [13]. In addition, salicylic acid, vanillic acid, protocatechuic acid, quercetin, ferulic acid, caffeic acid, and kaempferol were present as glycosides or glycosylated derivatives in the present study, and this form of presence was beneficial to enhance the antioxidant activity of quinoa [13,14]. Overall, nine samples could be clearly classified into three categories based on the hierarchical cluster analysis (HCA) (Figure 1D), which showed that the three distinct grain colors of quinoa differed in the expression of phenolic compounds.

### 2.3. Multivariate Statistical Analysis

**Figure 2:**
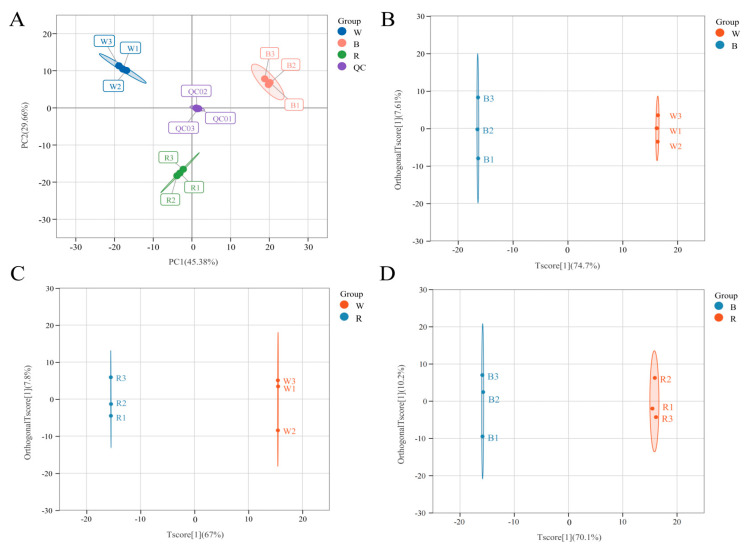
Principal component analysis (PCA) and orthogonal partial least squares discriminant analysis (OPLS-DA) results of polyphenol metabolites in white, black, and red quinoa seeds. (**A**) PCA shows metabolite profile differences between and within groups. OPLS-DA score plots of W vs. B groups (**B**), W vs. R groups (**C**), and B vs. R group (**D**).

As shown in Figure 2A, the extraction rates of two principal components (PC1 and PC2) in the PCA score plot were 45.38% and 29.66%, respectively. Three replicate QC samples were tightly clustered in the middle of the PCA plot, and the separation of the three samples was obvious, with the three biological replicate sequences of each sample forming a tight cluster, confirming the reproducibility and reliability of this experiment. The OPLS-DA model was employed for a two-by-two comparison of the three colored quinoa to detect the differences in polyphenol metabolites, with the scores of R^2^Y and Q^2^ of both surpassing 0.9 (Figure S3), demonstrating that the OPLS-DA model was suitable and predictable enough without overfitting. The three sets of paired samples could be easily distinguished by the OPLS-DA score plots (Figure 2B–D), showing that the samples with varied grain colors were statistically distinct and could be subjected to the identification of differential metabolites.

### 2.4. Differential Metabolic Polyphenol in Three Different Colored Quinoa

**Figure 3:**
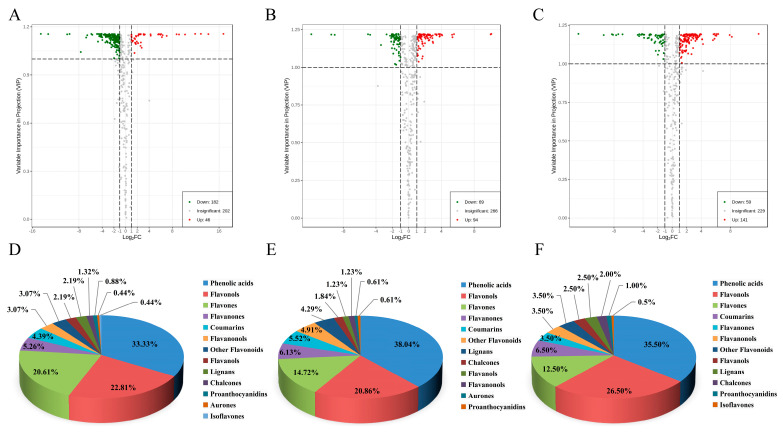
Differential polyphenol metabolite profiles and classification. (**A**–**C**) Volcano plots of differential phenolic compounds in white, black, and red quinoa seeds: (**A**) W vs. B; (**B**) W vs. R; (**C**) B vs. R. (**D**,**E**) Classification of differential polyphenol metabolites for three comparison groups: (**D**) W vs. B; (**E**) W vs. R; (**F**) B vs. R.

The volcano plots of different metabolites are shown in Figure 3A–C. There were 228, 163, and 200 species of significantly different phenolic metabolites in the W and B, W and R, and B and R comparisons, respectively. Of these significantly different phenolic metabolites, 182, 94, and 52 species of compounds showed decreased accumulation, while 46, 69, and 141 species of compounds showed increased accumulation in W and B, W and R, and B and R. The differential polyphenol metabolites between the three comparative groups mainly included phenolic acids, flavonols, and flavonoids (Figure 3D–F). The relative contents of phenolic acids and flavonoids were higher in W, flavonols and lignans were higher in R, and flavanols and proanthocyanidins were higher in B (Table S1). The results are basically consistent with the reports of Qian et al. [13]. Liu et al. found that most phenolic acids were higher in R than in B and W, which is not consistent with the present study [12]. Flavonoids, such as isoquercitrin, quercetin-3-*O*-rutinoside, luteolin-7-*O*-glucuronide, luteolin-7-*O*-neoconjugate, and kaempferol-3-*O*-rutinoside, are believed to be related to the color depth of quinoa seeds [12], but in our study, we found that the differentially expressed flavonoids were mostly more abundant in W and lower in B. That is to say, the color depth of quinoa seeds is not the result of the influence of a single type of polyphenol compound, but the result of the interaction of multiple chemical substances. Moreover, the polyphenol composition and content of quinoa might be related not only to grain color, but also to the variety and growth conditions of quinoa.

### 2.5. Key Differential Polyphenol Metabolite Screening

**Figure 4:**
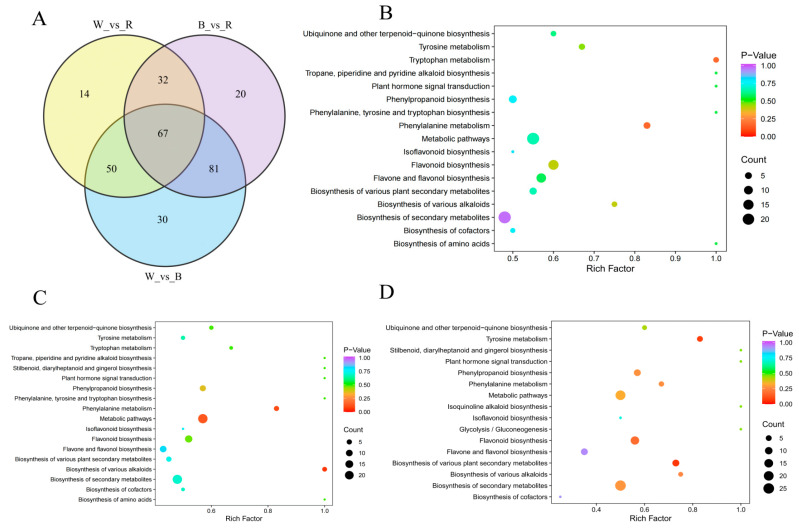
Identification and related metabolic pathways of key differential polyphenol metabolites in white, black, and red quinoa seeds. (**A**) Distribution of the key differential polyphenol metabolites in the three quinoa seeds. KEGG pathway analysis of differential metabolites for three comparison groups: (**B**) W vs. B; (**C**) W vs. R; (**D**) B vs. R.

Venn diagrams were further used to illustrate the relationships of phenolic metabolites among different colored quinoa (W/B/R) (Figure 4A). In a two-by-two comparison of three groups, 67 overlapping differential polyphenol metabolites were attributed to the key metabolites of colored quinoa, including 36 flavonoids, 23 phenolic acids, 7 lignans and coumarins, and 1 proanthocyanidin (Table S2), with flavonoids and phenolic acids accounting for 53.7% and 34.3% of the key differential metabolites. Ten substances with high contents were screened from the key difference compounds, among which epicatechin, naringenin, and proanthocyanidin B3 were the most abundant in the B, hesperidin-5-*O*-glucoside, quercetin-3-*O*-glucoside in the R, α-hydroxycinnamic acid, 3-hydroxycinnamic acid, 2-hydroxycinnamic acid, cinnamic acid, and 3-(4-hydroxyphenyl) propionic acid in the W groups. Studies have confirmed that epicatechin, quercetin-3-*O*-glucoside, naringenin, proanthocyanidin B3, and 3-(4-hydroxyphenyl) propionic acid have good antioxidant effects [15,16,17]. Hydroxycinnamic acid was found to be a chain-breaking antioxidant that can exert antioxidant effects by scavenging free radicals. In addition, hesperidin-5-*O*-glucoside with its OH group at C5 can inhibit xanthine oxidase activity and thus prevent the conversion of xanthine (XO) to uric acid, preventing the generation of superoxide radicals [18]. The above results suggest that different colored quinoa show significant differences in their characteristic phenolic composition and distribution, which may affect their potential biological activities such as antioxidant and hypoglycemic effects.

### 2.6. KEGG Pathway Annotation of the Differential Polyphenol Metabolites

The KEGG analysis showed that W and B, W and R, and B and R were involved in 17, 18, and 16 pathways, respectively (Figure 4B–D). The differential metabolite enrichment between W and B, and W and R, occurred mainly in phenylalanine metabolism and alkaloid biosynthesis; the differential metabolite enrichment between B and R occurred mainly in tyrosine metabolism, alkaloid biosynthesis, and flavonoid biosynthesis. Phenylalanine is the principal precursor ingredient for phenolic synthesis, which can create anthocyanins via the flavonoid metabolic branch and phenolic acids, including chlorogenic acid, caffeic acid, and *p*-coumaric acid via other branches, all of which have beneficial bioactive effects [19,20]. Tyrosine, a precursor of catecholamines, is involved in human energy metabolism and hormonal regulation and plays an important role in adrenal neurons [21]. Alkaloids, as a class of secondary metabolites of plants, are closely related to plant autoimmunity and resistance to adverse environmental effects. They have antitumor and antiviral effects in humans. Flavonoids are also secondary metabolites that protect plants from environmental stresses. They possess extensive health-beneficial activities, and their biosynthesis in plants is regulated by various enzymes including chalcone synthase (CHS), flavanone 3-hydroxylase/flavanol synthase (F3H), and dihydroflavonol-4-reductase (DFR) [22]. Taken together, the differential phenolic metabolism of colored quinoa mainly involves phenylalanine, tyrosine, and the biosynthesis of various plant secondary metabolites, which could contribute to the differences in the color of the quinoa seed coat and polyphenol activity.

### 2.7. Polyphenol Content and the In Vitro Antioxidant and Hypoglycemic Capacity of Different Colored Quinoa

**Figure 5:**
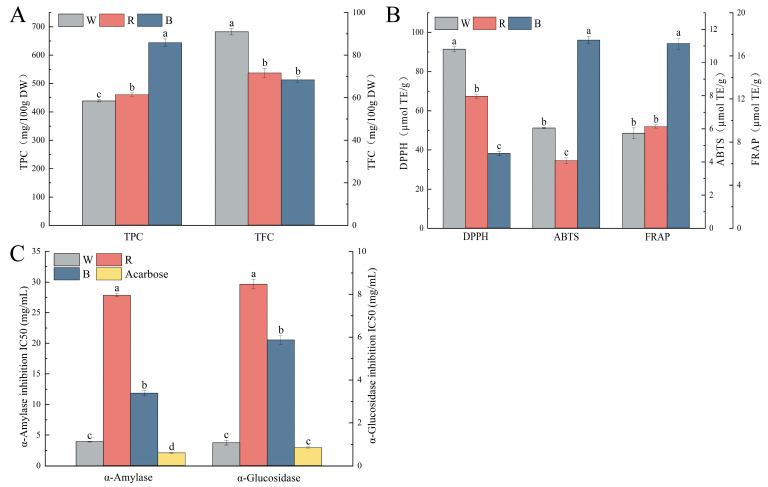
Total phenolic content (TPC), total flavonoid content (TFC), in vitro antioxidant and enzyme inhibition activities of phenolic extracts from white, black, and red quinoa seeds. (**A**) TPC and TFC content of the extracts. (**B**) DPPH, ABTS, and FRAP antioxidant capacity of the extracts. (**C**) IC50 values of α-amylase and α-glucosidase inhibition by the extracts (**C**). Note: DPPH: DPPH free radical scavenging activity; FRAP: ferric reducing power; ABTS: ABTS free radical scavenging activity. Different letters within the same group indicate significant differences (*p* < 0.05).

As shown in Figure 5A, there were significant differences in TPC and TFC among different colored quinoa. The TPC ranged from 438.75 to 643.68 mg/100 g DW, in the order of B > R > W, and the TFC ranged from 68.34 to 90.95 mg/100 g DW, in the order of W > R > B. The TPC tended to increase and the TFC tended to decrease as the seeds deepened in color. Liu et al. detected the TPC of three different varieties of quinoa (R, B, and W), and found that the TPC and TFC were the highest in R, followed by B, which was different from the present study [23]. The possible reasons could be differences in the extraction solvents and methods used, as well as the varieties of quinoa and their cultivation conditions.

The antioxidant activities are shown in Figure 5B. The DPPH radical scavenging capacity ranged from 38.11 to 91.49 µmol TE/g DW, in the order of W > R > B; the ABTS radical scavenging capacity ranged from 4.06 to 11.36 µmol TE/g DW, in the order of B > W > R; the FRAP reducing power ranged from 8.83 to 17.14 µmol TE/g DW, in the order of B > R > W. This is similar to the results of Ong et al. [24], but inconsistent with Liu et al.’s report [23]. This indicates that different varieties of quinoa and environmental conditions of cultivation may cause differences in the metabolism and accumulation of secondary polyphenol compounds, which leads to differences in polyphenol profiles and their antioxidant capacity.

As shown in Figure 5C, the polyphenol extracts from quinoa inhibited α-amylase and α-glucosidase both in the following order: W > B > R, and both were significantly lower than the positive control acarbose, which indicates the potential hypoglycemic activity of polyphenols of different colored quinoa. Han et al. studied the inhibitory effects of phenolic compounds from seven colored quinoa on α-glucosidase and found that quinoa polyphenols had good inhibitory activity toward α-glucosidase with an IC50 of 42.23–106.54 μg/mL, consistent with the results of this study [9]. In conclusion, the different colored quinoa had certain in vitro antioxidant activities and blood glucose regulation abilities, and the level of their activity varied significantly depending on the grain color and species of quinoa. Among them, B had a higher TPC and comprehensive in vitro antioxidant ability, while W had a higher TFC and in vitro hypoglycemic ability. It can be seen that the quinoa genotype had a significant effect on its potential bioactivity. This finding enriches our understanding of the impact of the health-promoting properties of colored quinoa polyphenols and provides a reference for their diversified development and utilization.

### 2.8. Correlation Analysis

To investigate the relationship between differential polyphenol metabolites and the in vitro antioxidant and enzyme inhibitory activities, we performed a correlation analysis and screened key phenolic compounds. As depicted in Table 1, the TPC correlated positively with ABTS and FRAP, whereas the TFC correlated positively with DPPH, findings that are consistent with those published by Li et al. [19]. The correlation coefficients between 67 key differential compounds and antioxidant activity are presented in Table 1. There was a significant positive correlation between 21 monomeric phenols and DPPH, of which cinnamic acid, 2-hydroxycinnamic acid, 3-hydroxycinnamic acid, and α-hydroxycinnamic acid are known to have antioxidant effects, and they all had the highest content in W, which may be the key to the strongest DPPH free radical scavenging capacity of W polyphenols.

Additionally, 15 phenolic substances were positively linked with ABTS and FRAP (Table 1), of which epicatechin, naringenin, and proanthocyanidin B3 are known to have antioxidant effects, and they all had the highest content in B, which may be the key to the strongest ABTS and FRAP antioxidant capacities of B polyphenols. In addition, epicatechin, naringenin, and proanthocyanidin B3 might not only be closely related to polyphenol antioxidant activity but also to color [17,25,26].

Table 1 further shows that both α-amylase and α-glucosidase inhibition rates, as evaluated by their respective IC50 values, were positively related to the TFC, implying that a higher TFC leads to better hypoglycemic effectiveness. Six monomeric phenols, including kaempferol-3-*O*-(2″-*O*-acetyl) glucuronide, kaempferol-3-*O*-(6″-*p*-coumaryl) glucoside, cinnamic acid, chrysoserin, kaempferol, and isofraxidin, were significantly negatively correlated with the IC50 value, speculating that they may play a crucial role in decreasing blood sugar. Han et al. investigated quinoa polyphenols and discovered that quercetin, vanillin, and gallic acid had a strong linear correlation with the α-glucosidase inhibition rate [9]. Hassan et al. confirmed that ferulic acid, resveratrol, quercetin, caffeic acid, epicatechin, and naringin could significantly inhibit α-glucosidase activity. Moreover, catechins, hesperidin, kaempferol, and naringenin could significantly inhibit α-amylase activity [27]. It can be seen that the inhibitory action of polyphenols on α-glucosidase and α-amylase is not produced by a single component but rather by the cumulative and synergistic effects of numerous monomeric phenolic compounds. To sum up, there was a significant correlation between the phenolic compounds of colored quinoa and their in vitro antioxidant and enzyme inhibitory activities, in which 36 monomeric phenols were key to the in vitro antioxidant capacity of quinoa polyphenols (Table S3), while 6 monomeric phenols were key to the in vitro glucose regulation capacity of quinoa polyphenols (Table S4).

## 3. Materials and Methods

### 3.1. Chemicals and Materials

The varieties of white quinoa (Daibaili, W), red quinoa (Gongzha No. 4, R), and black quinoa (Qingli No. 2, B) were supplied by Qinghai Academy of Agriculture and Forestry Sciences (Xining, China) and planted in 2022 in the experimental field (36°67′ N, 101°77′ E, altitude 2300 m) under identical soil fertility, cultivation, and meteorological circumstances. The test material was stored at −80 °C after hulling until metabolomic analysis and in vitro activity analysis. 1,1-Diphenyl-2-picrylhydrazyl (DPPH), 2,4,6-tripyridyl-s-triazine (TPTZ), 2,20-azinobis-(3-ethylbenzthiazoline-6-sulfonate) (ABTS), Trolox (water-soluble vitamin E), and *p*-nitrophenol glucopyranoside (PNPG) with BR level were supplied by Sigma-Aldrich (St. Louis, MO, USA). Folin–Ciocalteu (GR level), acarbose (purity ≥ 95%), and 3,5-dinitrosalicylic acid (DNS) were supplied by Beijing Solarbio Science & Technology Co., Ltd. (Beijing, China). The α-amylase (10 U/mg) from porcine pancreas and α-glucosidase (50 U/mg) from yeast were purchased from Shanghai Yuanye Bio-Technology Co., Ltd. (Shanghai, China). Sodium carbonate, sodium hydroxide, potassium chloride, sodium acetate, sodium nitrite, aluminum nitrate, ferric chloride, potassium persulphate, disodium hydrogen phosphate, sodium dihydrogen phosphate, and concentrated HCl with AR level were supplied by Tianjin Hengxing Chemical Reagent Manufacturing Co., Ltd. (Tianjin, China). Acetone and methanol of chromatographic grade were provided by Tianjin Fuyu Fine Chemical Co., Ltd. (Tianjin, China). Deionized water was used throughout the experiment.

### 3.2. Sample Preparation

Quinoa specimens were vacuum-freeze-dried (Scientz-100F, Hangzhou Deju Instruments Co., Ltd., Hangzhou, China) before being ground for 1.5 min at 30 Hz on a grinder (MM 400, Retsch, Haan, Germany). About 50 mg of the pulverized powder was weighed, dissolved in 1.2 mL of a 70% methanol extract, and vortexed every 30 min for a total of 6 times. After centrifuging at 12,000 rpm for 3 min, the supernatant was aspirated, filtered through a microporous membrane with a 0.22 µm pore size, and then placed in an injection vial for UPLC–MS/MS analysis. Each quinoa species underwent three distinct biological replicates. A variety of extracts from samples of various grain colors made up the quality control (QC) samples.

### 3.3. Chromatography and Mass Spectrometry Conditions

A UPLC–MS/MS system equipped with an ExionLC^TM^ AD chromatography system and an Applied Biosystems 4500 QTRAP mass spectrometry system (SCIEX, Framingham, MA, USA) was used for metabolite analysis. The column used in the UPLC was an Agilent SB-C18 (1.8 µm, 2.1 mm × 100 mm) column. Mobile phase A was ultrapure water containing 0.1% formic acid, whereas mobile phase B was acetonitrile with 0.1% formic acid. The elution gradient was as follows: 0–9 min, 5–95% B; 10–11 min, 95–5% B; 12–14 min, 5% B. The sample injection volume was 4 µL. The column temperature was 40 °C and the flow rate was 0.35 mL/min. To reduce analytical bias, samples were injected in completely random order. The mass spectrometry conditions were as follows: The electrospray ionization source (ESI) temperature and ion spray voltage (IS) were 550 °C and 5500 V (positive ionization mode)/−4500 V (negative ionization mode), respectively. The ionization source gas I (GSI), gas II (GSII), and curtain gas (CUR) were set to 50, 60, and 25 psi, respectively, and the collision-induced ionization parameters were set to high. With the collision gas (nitrogen) set to medium, QQQ scans were performed in MRM mode. Further declustering potential (DP) and collision energy (CE) tuning for specific MRM ion pair DP and CE were completed. Depending on the metabolites that were eluted at each interval, a particular set of multiple reaction monitoring (MRM) ion pairs was observed.

### 3.4. Metabolite Qualitative and Quantitative Analysis

After gathering the metabolite spectrum analysis data of multiple samples and integrating the peak areas of all substance peaks, the mass spectra of the same metabolite in different samples were corrected for peak integration. Secondary MS data from the self-built database metware database (MWDB) were used to characterize substances with reference to public databases (METLIN, HMDB, ChemBank, PubChem, and MassBank). MRM mode of triple-quadrupole MS was used for the metabolite quantification. In MRM mode, the quadrupole initially selects the precursor ions (parent ions) of the target substances, thereby eliminating ions corresponding to other molecular weights to preliminarily remove interferences; the precursor ions, after being induced to ionize in the collision chamber, fragment into multiple fragment ions. These fragment ions are then filtered through the triple quadrupole to select a characteristic fragment ion, eliminating non-target ion interference, thus rendering the quantification more precise and with better repeatability. After obtaining the metabolite spectrum analysis data of multiple samples, the mass spectra peak areas of all compounds were integrated using the MultiQuant version 3.0.2 (AB Sciex, Framingham, MA, USA). Finally, the relative contents of the corresponding metabolites were expressed as the chromatographic peak area integrals.

### 3.5. Statistical Analysis

After eliminating chemicals with high deviations (CV values greater than 0.5), the models’ stability and reliability were predicted using principal component analysis (PCA), cluster analysis, partial least squares discriminant analysis (PLS-DA), and orthogonal partial least squares discriminant analysis (OPLS-DA). After screening all metabolites for orthogonal variables, the OPLS-DA model was validated using 200 random permutations. Metabolites with VIP ≥ 1, *p* < 0.05, FC ≥ 2, and FC ≤ 0.5 were chosen as the differential metabolites. The various metabolites in each category were grouped hierarchically. The KEGG compound database (http://www.kegg.jp/kegg/compound/, accessed on 22 March 2024) was used to annotate metabolites, which were then mapped to the KEGG pathway database (http://www.kegg.jp/kegg/pathway.html, accessed on 22 March 2024). The metabolite enrichment analysis (MSEA) was performed for pathways with significant regulation. *p*-values from hypergeometric distribution tests were used to determine significance.

### 3.6. Phenolic Content Determination

#### 3.6.1. Polyphenol Extracts Preparation

The polyphenol extracts of colored quinoa were prepared according to Zhang et al.’s method [28]. Briefly, 1 g of quinoa flour was weighted and mixed with 25 mL of 80% acetone. The mixture was then subjected to ultrasonic treatment at 100 Hz at room temperature for 30 min, followed by centrifugation (4000 r/min, 10 min) to collect the supernatant. The residue was extracted twice more using the same procedure, and the three supernatants were combined. The combined extracts were then dried under reduced pressure at 45 °C using a rotary evaporator. The residue was reconstituted to a final volume of 10 mL with methanol and filtered through a 0.45 µm organic membrane to obtain the free phenolic compound extract of quinoa. The extract was stored at −20 °C, protected from light.

#### 3.6.2. Total Phenolic Content

The total phenolic content (TPC) of the polyphenol extracts was detected using the Folin–Ciocalteu method [28]. In short, 125 µL of the sample extract was transferred into a test tube, to which 500 µL of distilled water and 125 µL of Folin–Ciocalteu reagent were added. The mixture was vortexed, allowed to react for 6 min, and then added to 1.25 mL of 7% Na_2_CO_3_ solution and 1 mL of distilled water. The solution was then left to stand in the dark at room temperature for 1.5 h. The absorbance was measured at a wavelength of 760 nm, and the TPC in the samples was calculated based on the standard curve and expressed in terms of gallic acid equivalents (mg GAE/100 g DW).

#### 3.6.3. Total Flavonoid Content

The total flavonoid content (TFC) of the polyphenol extracts was detected using the colorimetric method [28]. Briefly, 100 µL of quinoa free phenolic extracts were dispensed into a test tube, followed by the addition of 200 µL of 5% NaNO_2_ solution. The mixture was vortexed, left to react for 6 min, and then added to 200 µL of 10% Al(NO_3_)_3_ solution. After an additional 6 min of reaction, 2 mL of 4% NaOH solution and 2.5 mL of distilled water were added. The mixture was then set aside in the dark at room temperature for 15 min. The absorbance was measured at a wavelength of 510 nm, and the TFC of the samples was calculated based on the standard curve and expressed in terms of rutin equivalents (µmol/100 g DW).

### 3.7. Antioxidant Capacity Determination

#### 3.7.1. DPPH Free Radical Scavenging Activity

The DPPH free radical scavenging capacity of the extract was determined according to the previous literature [29]. In short, 200 µL of the polyphenol extract was mixed with 4.5 mL of 0.1 mmol/L DPPH–methanol solution. After reacting for 30 min in the dark, the sample absorbance was measured at 517 nm. DPPH free radical scavenging activity was represented as Trolox equivalents (µmol TE/100 g DW).

#### 3.7.2. Ferric Reducing Antioxidant Power

The ferric reducing antioxidant power (FRAP) of the extract was detected according to the literature [29]. In short, 200 µL of the polyphenol extract was shaken well with 4.5 mL of FRAP working solution and then reacted for 30 min in the dark. The final sample absorbance was measured at 593 nm. The FRAP antioxidant capacity was represented as Trolox equivalents (µmol TE/g DW).

#### 3.7.3. ABTS Free Radical Scavenging Activity

ABTS free radical scavenging capacity of the extract was determined based on the literature [29]. Briefly, 200 µL of the polyphenol extract was shaken well with 4 mL ABTS free radical reserve solution, and then reacted for 30 min in the dark. The absorbance was measured at 734 nm. The ABTS antioxidant capacity was represented as Trolox equivalents (µmol TE/g DW).

### 3.8. In Vitro Hypoglycemic Activity Determination

#### 3.8.1. α-Amylase Inhibition Rate

The α-amylase inhibitory activity of the extract was detected according to Jin et al.’s method and the acarbose was used as a positive control [30]. In brief, 500 µL of the polyphenol extract and an equivalent amount of the α-amylase solution (2.0 U/mL) were combined equally and incubated at 37 °C for 10 min before 500 µL of the 1% soluble starch solution was added. The reaction was terminated by adding 1 mL of the color indicator DNS reagent, followed by a 5 min boiling water bath. The mixture was diluted with 10 mL of distilled water and the sample absorbance was measured at 540 nm. The following equation was used to calculate the α-amylase inhibition rate (1).

(1) $$\alpha -Amylase\;inhibition\;rate=1-\frac{A_{1}-A_{2}}{A_{3}}\times 100\%$$

In the equation:

A_1_ is the sample absorbance value (sample solution + α-amylase solution + 1% soluble starch solution + DNS reagent + distilled water).

A_2_ is the background absorbance value (sample solution + buffered solution + 1% soluble starch solution + DNS reagent + distilled water).

A_3_ is the blank absorbance value (buffered solution + α-amylase solution + 1% soluble starch solution + DNS reagent + distilled water).

#### 3.8.2. α-Glucosidase Inhibition Rate

The α-glucosidase inhibitory activity of the extract was detected according to Jin et al.’s method and the acarbose was used as a positive control [30]. In brief, in each well of a 96-well plate, 40 μL of the polyphenol extract and 30 µL of α-glucosidase solution (0.2 U/mL) were added and mixed thoroughly. The mixture was then incubated at 37 °C for 10 min, followed by the addition of 30 µL of PNPG (5 mmol/L) to each well. After thorough mixing, the plate was incubated again at 37 °C for 30 min. The reaction was halted by adding 100 µL of Na_2_CO_3_ (1 mol/L), and the absorbance A was measured at 405 nm. The following equation was used to calculate the α-glucosidase inhibition rate (2).

(2) $$\alpha -Glucosidase\;inhibition\;rate=1-\frac{A_{1}-A_{2}}{A_{3}}\times 100\%$$

In the equation:

A_1_ is the sample absorbance value (sample solution + α-glucosidase solution + PNPG solution + Na_2_CO_3_ solution).

A_2_ is the background absorbance value (sample solution + α-glucosidase solution + buffered solution + Na_2_CO_3_ solution).

A_3_ is the blank absorbance value (buffered solution + α-glucosidase solution + PNPG solution + Na_2_CO_3_ solution).

## 4. Conclusions

This study investigated the expression differences of phenolic compounds of white, black, and red *Chenopodium quinoa* Willd. using the UPLC–MS/MS-based widely targeted metabolomics approach. The relationship between the relevant differential metabolites and the in vitro antioxidant and hypoglycemic activities of quinoa polyphenols was further evaluated. A total of 430 polyphenols were discovered in colored quinoa, mostly including phenolic acids, flavonoids, and flavonols, and 67 phenolic compounds were identified as significant differential metabolites. Phenylalanine metabolism, tyrosine metabolism, and the biosynthesis of various plant secondary metabolites are the key pathways to regulate the formation of differential polyphenols in quinoa with different grain colors. Black quinoa had a better total phenolic level and antioxidant capacity, while white quinoa had a better total flavonoid level and in vitro hypoglycemic activity. It was found that the grain color of quinoa seemed to be the result of various compounds and their interactions, and the grain color depth was not the only factor that determined the activity of quinoa polyphenols. Based on the correlation analysis, 36 monomeric phenols were selected as the main quinoa polyphenols contributing to antioxidant capacity and 6 monomeric phenols were determined to be the main quinoa polyphenols contributing to the inhibition of α-amylase and α-glucosidase activities. In the present work, three colored quinoa grown in the same conditions were used. However, the accumulation differences and biological activities of characteristic quinoa phenolic compounds from large sample populations and under different growing conditions are worth further exploration. Overall, this research is significant for investigating the usefulness of quinoa polyphenols as well as the creation and usage of colored quinoa resources in health foods.

## Figures and Tables

**Table 1:** Pearson correlation coefficients between total phenolics, total flavonoids, and 67 key differential polyphenol metabolites and the extracts’ in vitro antioxidant and α-amylase and α-glucosidase inhibitory activities.

Compounds	DPPH	ABTS	FRAP	α-Amylase IC50	α-Glucosidase IC50
Total phenolics		0.933 **	0.989 **	−0.099	0.261
Total flavonoids	0.848 **	−0.355	−0.625	−0.628	−0.842 **
Lariciresinol-4′-*O*-glucoside	0.878 **				−0.881 **
Isorhamnetin-3-*O*-arabinoside	0.930 **				−0.775 *
Chrysoeriol-6-C-rhamnoside-7-*O*-rhamnoside	0.948 **				−0.761 *
Luteolin-7-*O*-(6″-caffeoyl)rhamnoside	0.791 *				−0.842 **
Methyl 2,4-dihydroxyphenylacetate	0.894 **				−0.713 *
(2E)-3-[4-(β-D-glucopyranoside)-phenylacrylic]-acid	0.931 **				−0.802 **
α-Hydroxycinnamic Acid	0.929 **				−0.776 *
3-Hydroxycinnamic Acid	0.959 **				−0.735 *
Salicylic acid		0.955 **	0.982 **		
2-Hydroxycinnamic acid	0.866 **				−0.879 **
Kaempferol-3-*O*-(6″-*p*-Coumaroyl)galactoside	0.932 **				−0.79 *
3-(4-Hydroxyphenyl)-propionic acid	0.909 **				−0.844 **
3,5,7-Trihydroxyflavanone		0.983 **	0.867 **		
*p*-Coumaraldehyde		0.950 **	0.994 **		
Tiliroside	0.877 **			−0.669	−0.889 **
Isofraxidin				−0.935	−0.974 **
Cinnamic acid	0.788 *			−0.785	−0.953 **
4-Hydroxyphenylethanol		0.945 **	0.996 **		
4-Hydroxyphenyllactic Acid	0.894 **				−0.713 *
Caffeic aldehyde		0.987 **	0.971 **		
Kaempferide	0.761 *			−0.678	−0.854 **
Isosakuranetin-7-*O*-glucoside	0.912 **				−0.843 **
Epicatechin		0.955 **	0.988 **		
Scopoletin-7-*O*-glucoside	0.907 **				−0.827 **
3,4-Dihydrocoumarin		0.951 **	0.976 **		
2,3,4-Trihydroxybenzoic acid	0.934 **				−0.794 *
Disinapoyl glucoside		0.793 *	0.925 **		
Catechin-catechin-catechin		0.963 **	0.990 **		
Naringenin		0.981 **	0.865 **		
Procyanidin B3		0.962 **	0.981 **		
Phloretin		0.938 **	0.998 **		
Homogentisic acid		0.933 **	0.989 **		
3,4-Dihydroxybenzeneacetic acid		0.943 **	0.987 **		
Kaempferol-3-*O*-(2″-*O*-acetyl)glucuronide				−0.905 **	−0.979 **
Methyl sinapate		0.894 **	0.974 **		
Chrysoeriol	0.838 **			−0.719 *	−0.916 **
Acacetin-7-*O*-rutinoside	0.938 **				−0.778 *
2-*O*-Feruloylglucaric Acid	0.883 **				−0.856 **
9,11-dimethoxy-2h-[1,3]dioxolo[4,5-b]xanthen-10-one				−0.882 **	−0.967 **
8,11-dimethoxy-2h-[1,3]dioxolo[4,5-b]xanthen-10-one	0.859 **			−0.672 *	−0.886 **
6,7-Dimethoxy-4-chromanone		0.878 **	0.970 **		
Kaempferide-3-*O*-(6″-malonyl)glucoside	0.885 **				−0.871 **
Robinson-7-*O*-Neohesperidin	0.926 **				−0.778 *

Note: DPPH: DPPH free radical scavenging activity; FRAP: ferric reducing power; ABTS: ABTS free radical scavenging activity; ** indicates significance *p* < 0.01.* indicates significance *p* < 0.05.

## Data Availability

Data are contained within the article and supplementary materials.

## Supplementary Materials

The following supporting information can be downloaded at: https://www.mdpi.com/article/10.3390/molecules29071509/s1. Table S1: List of identified metabolites between quality control sample, white, black, and red quinoa seeds. Table S2: 67 key differential polyphenol contents in white, black, and red quinoa seeds. Table S3: List of 36 metabolites associated with in vitro antioxidant capacity. Table S4: List of 6 metabolites associated with in vitro glucose regulation capacity. Figure S1: Overlapping diagram of total ion current (TIC) maps from mixed QC sample mass spectrometry in (A) negative ion mode and (B) positive ion mode. Multi-peak detection plots of metabolites acquired in (C) negative ion mode and (D) positive ion mode. Figure S2: Variation of the relative abundance of 30 selected phenolic compounds among the three quinoa seeds. Figure S3: Variable value R^2^X and predictability value Q^2^ of principal component analysis (PCA) model.
