# Supplementary material for: Metabolomics Characterization of Phenolic Compounds in Colored Quinoa and Their Relationship with In Vitro Antioxidant and Hypoglycemic Activities
Source: Molecules. 2024 Mar 28;29(7):1509. doi: 10.3390/molecules29071509 (PMC11013001; doi:10.3390/molecules29071509)
Supplement: Supplementary file 1 [file molecules-29-01509-s001.zip › Supplementary Figures File.pdf]

Metabolomics characterization of phenolic compounds in colored quinoa and their relationship with in vitro antioxidant and hypoglycemic activities

Ling Zhang [1], Bin Dang [1,2], Yongli Lan [3], Wancai Zheng [1,2], Jiwei Kuang [1,2], Jie Zhang [1,2] and Wengang Zhang [1,2,*]

[1] Laboratory for Research and Utilization of Qinghai Tibet Plateau Germplasm Resources, Qinghai University, Xining 810016, China; 13897604262@163.com (L.Z.); 2008990019@qhu.edu.cn (B.D.); 13565849218@163.com (W.C.Z.); 2023990011@qhu.edu.cn (J.K.); 2015990070@qhu.edu.cn (J.Z.)

[2] Key Laboratory of Qinghai Province Tibetan Plateau Agric-Product Processing, Qinghai University, Xining 810016, China

[3] College of Food Science and Engineering, Northwest A & F University, Yangling 712100, China; yonglilan@nwsuaf.edu.cn (Y.L.)

* Correspondence: 2017990098@qhu.edu.cn (W.G.Z.)

Figure captions:

Figure S1. Overlapping diagram of total ion current (TIC) maps from mixed QC sample mass spectrometry in (A) negative ion mode and (B) positive ion mode. Multi-peak detection plots of metabolites acquired in (C) negative ion mode and (D) positive ion mode.

Figure S2. Variation of the relative abundance of 30 selected phenolic compounds among the three quinoa seeds.

Figure S3. Variable value R^2X and predictability value Q^2 of principal component analysis (PCA) model.

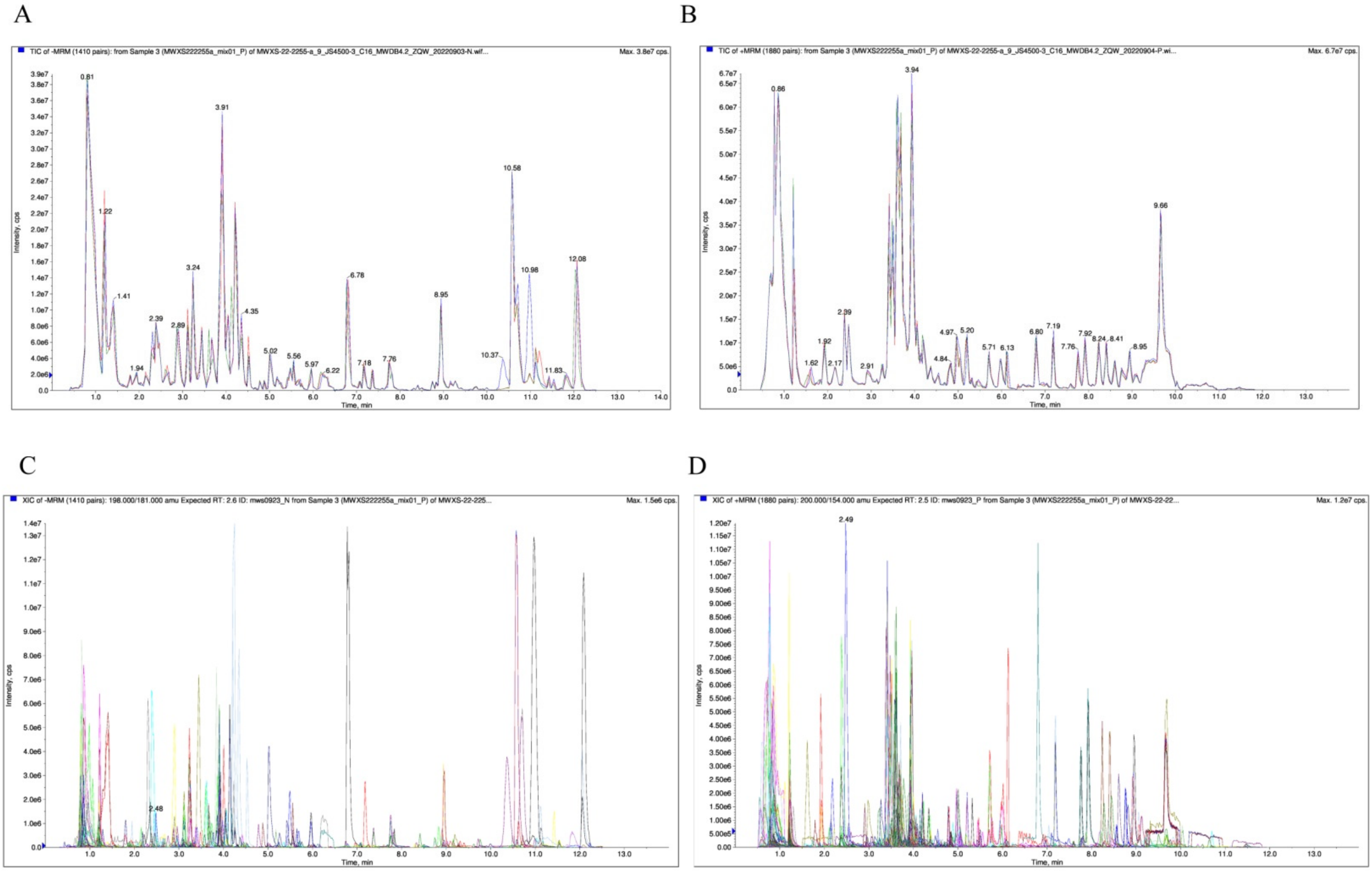

Figure S1. Overlapping diagram of total ion current (TIC) maps from mixed QC sample mass spectrometry in (A) negative ion mode and (B) positive ion mode. Multi-peak detection plots of metabolites acquired in (C) negative ion mode and (D) positive ion mode.

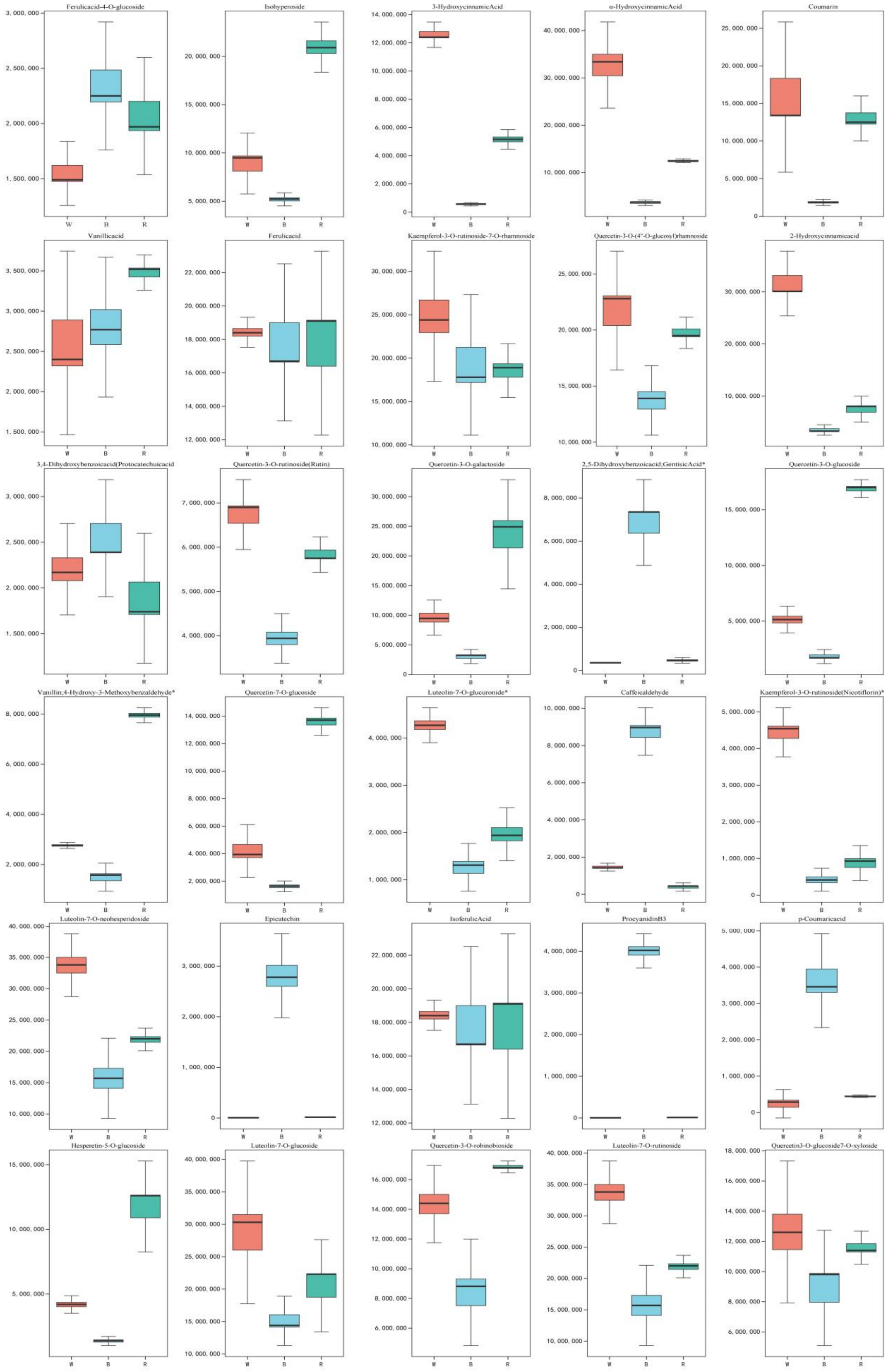

Figure S2. Variation of the relative abundance of 30 selected phenolic compounds among the three quinoa seeds.

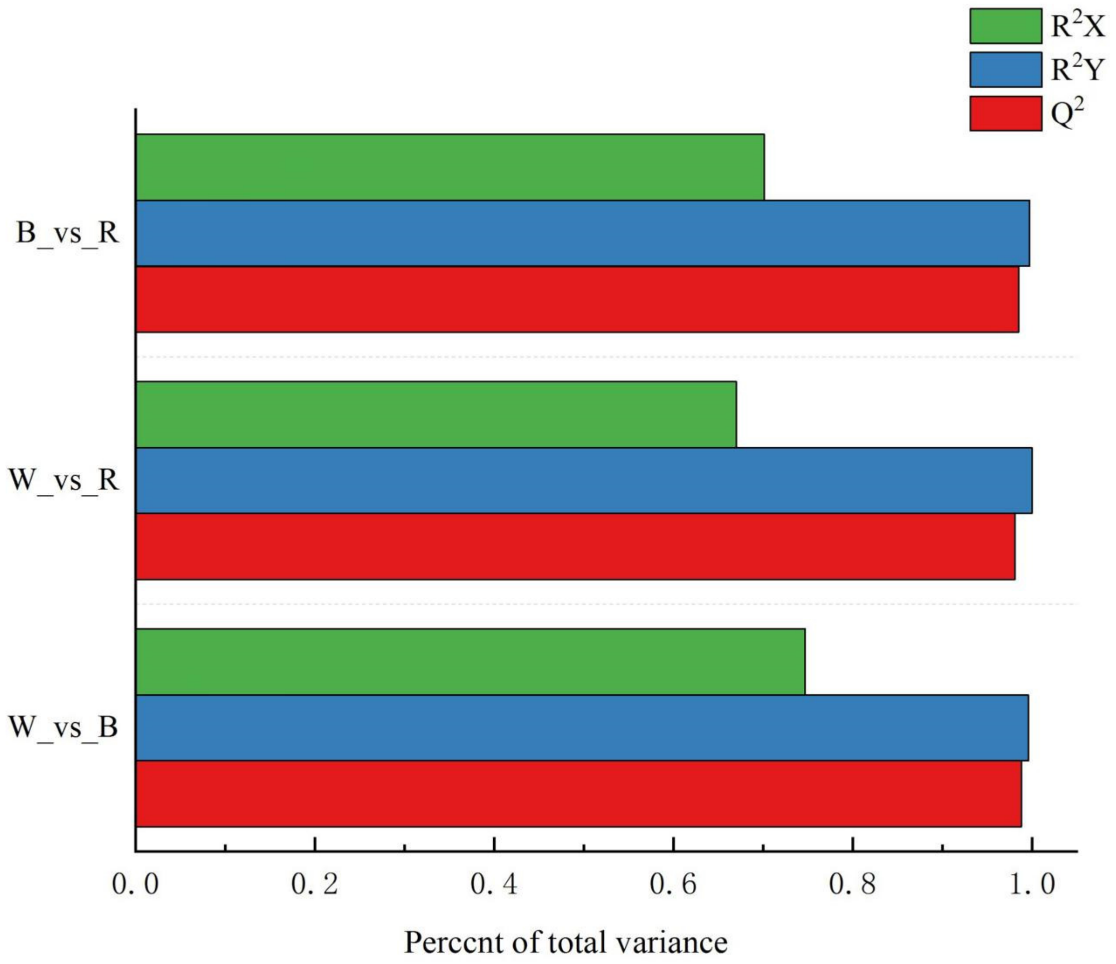

Figure S3. Variable value R^2X and predictability value Q^2 of principal component analysis (PCA) model.
